# Eco-friendly 2-Thiobarbituric acid as a corrosion inhibitor for API 5L X60 steel in simulated sweet oilfield environment: Electrochemical and surface analysis studies

**DOI:** 10.1038/s41598-018-37049-w

**Published:** 2019-01-29

**Authors:** Bashir J. Usman, Zuhair M. Gasem, Saviour A. Umoren, Moses M. Solomon

**Affiliations:** 1 Mechanical Engineering Department, King Fahd University of Petroleum and Minerals, Dhahran, Saudi Arabia; 2 Center of Research Excellence in Corrosion, Research Institute, King Fahd University of Petroleum and Minerals, Dhahran, Saudi Arabia

**Keywords:** Surface spectroscopy, Scanning electron microscopy

## Abstract

The corrosion inhibition efficiency of 2-Thiobarbituric acid (TBA) for metal substrate (API X60 steel) in 3.5% NaCl solution saturated with CO_2_ gas was probed using various techniques namely, LPR (linear polarization resistance), EIS (electrochemical impedance spectroscopy), and PDP (potentiodynamic polarization). The effects of TBA concentration (25–100 ppm), solution pH (4 and 6), temperature (25–80 °C), and immersion time (2–72 h) on the inhibition efficiency were examined. SEM (scanning electron microscopy) and XPS (X–ray photoelectron spectroscopy) were deployed to explore the corrosion retardation mechanism. TBA exhibited protection efficiencies exceeding 90% for all experimental conditions considered. The excellent anticorrosion performance by TBA was retained up to 72 hours of immersion time. PDP results exhibited that TBA behaved as a mixed type inhibitor. Results from kinetics and thermodynamics analyses indicate that TBA chemically adsorbed on the steel surface following Langmuir isotherm model. The composition of the adsorbed TBA film has been analyzed by XPS.

## Introduction

CO_2_ corrosion is a serious challenge faced in the oil and gas industries. Crude oil wells contain varying amounts of CO_2_ as an associate gas. CO_2_ gas readily dissolves in the formation water and forms a weak carbonic acid which leads to severe corrosion attack. Beside the general corrosion, CO_2_ also causes localized corrosion and as it is known, this type of corrosion is difficult to predict, detect, and protect against^1^. It is ranked as the topmost type of attack encountered in the production and transportation of crude oil^1,2^. In oilfields, 60% of failures are believed to be caused by corrosion and CO_2_ corrosion is the major contributor^3^.

The use of corrosion inhibitors is the most practical and less expensive approach widely employed to control corrosion in the oil and gas industries^4–8^. Hitherto, arsenates, chromates, ferrocyanide, and metavandate were the choice corrosion inhibitors for the oilfield corrosion but have long lost patronage on the basis of their high toxicity. As replacement, organic adsorption inhibitors such as imidazolines and their derivatives are used^7^. Another category of organic inhibitors in use in industrial formulations are products formed from the condensation reactions of organic compounds with amino, carbonyl, and hydroxyl functional groups^8^. The π-electrons and heteroatoms like N, O, P, and S present in the structures of organic compounds serve as adsorption centers and facilitate adsorption on substrate surface^6,7,9^. Nevertheless, some of these synthetic compounds are only effective at high concentrations and also possess toxicity that is inimical to the environment. The research focus of the present is on developing effective ecological friendly inhibitors – the so called green corrosion inhibitors that could take the place of organic and inorganic inhibitors^10^. The targeted class of compounds include natural polymers, plant extracts, amino acids, expired drugs, and medicinal products^11,12^.

Barbiturates are non-toxic central nervous system depressants and also effective as anxiolytics, hynotics, and anti-convulsants. Manufacturing industries such as plastics, polymers, textiles *etc*. are also common users of barbiturates^13^. There are few published works on the corrosion inhibition effect of barbiturates. It had been reported as a corrosion inhibitor for low carbon steel in HCl environment^13^. Ozcan *et al*.^14^ studied the anticorrosion performance of TBA, 5,5-diethylbarbituric acid sodium salt (DEBA), and barbituric acid (BA) for mild steel in 1 M H_3_PO_4_ medium^14^ and were found effective. BA, TBA, and ethyl barbituric acid (EBA) had equally been documented to be effective in suppressing mild steel dissolution in 0.5 Μ HCl environment^15^. To the best of our knowledge, there is no report on the use of barbiturates as inhibitors to protect steel against corrosion in CO_2_ saturated environments.

**Figure 1:**
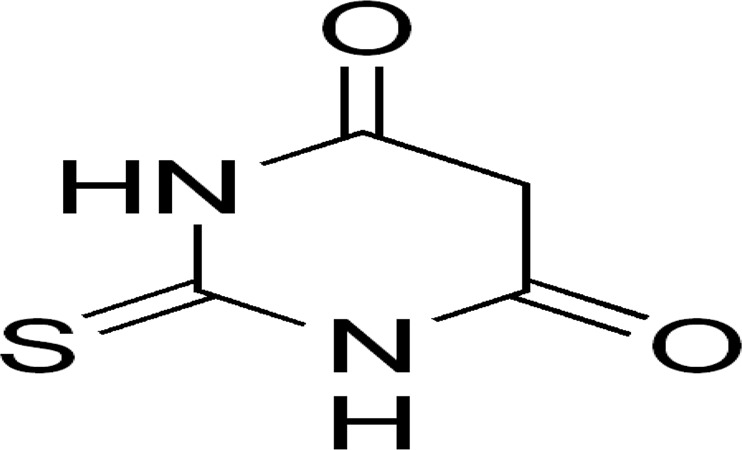
Chemical structure of 2- Thiobarbituric acid (TBA).

The present work aims to evaluate the performance of TBA as a corrosion inhibitor for API 5 L X60 steel in CO_2_-saturated NaCl medium via LPR, EIS, PDP, SEM, XPS, and FTIR techniques. The effect of pH and temperature has also been studied. We had earlier reported Tannic acid as ecofriendly corrosion inhibitor for the same metal substrate (API 5 L X60 steel) and the same corrosive environment^16^. TBA (Fig. 1) is a highly potential green inhibitor owing to the presence of N, O and S heteroatoms in its chemical structure in comparison to other barbiturates^17^. Also, TBA contains S heteroatom in addition to N and O which are present in all barbiturates. The additional S heteroatom in TBA could convey greater corrosion inhibition effect because of improved adsorption of the heteroatoms on metal surfaces in the order S > N > O^5^.

## Experimental

### Sample Preparation

An API 5 L X60 steel was used as the working electrode. The elemental composition is given in Table 1. A flat sheet of the metal was cut into 1 × 1 cm^2^ samples and were mounted in epoxy resin. The exposed surface (1 cm^2^) was abraded with different grit of SiC paper, washed with ethanol and distilled water, and then dried with warm air.

**Table 1:** Chemical composition of API X60 steel (wt.%).

C	Si	Mn	P	S	Cu	Mo, Ni	Cr,V	Fe
0.20	0.36	1.16	0.01	<0.003	0.18	0.10	0.08	Balance

### Solution Preparation

The corrosive medium was 0.6 M (3.5 wt.%) NaCl solution. The electrolyte solution was used to prepare different concentrations of TBA (Sigma Aldrich).

### Electrochemical testing

The electrochemical measurements were done following the ASTM G3–89^18^ and G5–94^19^ standard. The experiments were carried out making use of a Gamry potentiostat/galvanostat/ZRA (Reference 3000, Gamry) instrument. The counter electrode was a graphite rod while a saturated calomel electrode (SCE) play the role of a reference electrode. To mimic an oxygen free oilfield condition, N_2_ (99.99%) was bubbled into the solution for 30 minutes^20,21^. Thereafter, the environment was saturated with CO_2_ gas for 7200 s before the insertion of the working electrode. The pH was about 3.8 when the solution was saturated with CO_2_ and was adjusted to the working pH of 4.0 and 6.0 by addition of sodium bicarbonate (NaHCO_3_). The CO_2_ gas was bubbled continuously throughout the duration of experimental measurements.

In order to satisfy the essential requirement of stationarity, 3000 s were allowed for OCP (open circuit potential) stability prior to electrochemical testing. The EIS measurements were recorded at a sinusoidal excitation ac voltage of ±10 mV (rms) and the responses assessed in the frequency range of 10 mHz-100 kHz with ten points per decade. For LPR experiments, the API 5 L X60 steel electrode was polarized at ±15 mV relative to open circuit potential (E_ocp_) and the scan rate was 0.2 mVs^−1^. PDP measurements were achieved by polarizing the API 5 L X60 steel electrode at a potential range of ±250 mV vs. E_ocp_ at a rate of 0.2 mVs^−1^. For the purpose of reproducibility, each experiment was conducted in triplicate and average values of the electrochemical parameters are reported. The standard deviation computed for all the electrochemical parameters were less than 5% pointing to high reproducible data.

To quantitatively assess the anticorrosive ability of TBA, the percentage protection efficacy (%IE) was computed using Eqs (1–3) from EIS, LPR and PDP experiments respectively:

1 $$ \% IE=\,\frac{{R}_{cti-{R}_{ctb}}}{{R}_{ctb}}\times 100$$

where R_cti_ and R_ctb_ are the charge transfer resistances in presence and absence of TBA, respectively.

2 $$ \% IE=\frac{{R}_{p,i}-{R}_{p,b}}{{R}_{p,i}}\ast 100$$

where R_p,i_ and R_p,b_ are the polarization resistances of the specimen in the corrosive system without and with TBA, respectively.

3 $$ \% IE=\frac{{i}_{corr,b}-\,{i}_{corr,i}\,}{{i}_{corr,b}}\ast 100$$

where i_corr,b_ and i_corr,i_ are the corrosion current densities in the absence and presence of TBA, respectively.

### Surface screening

SEM (JOEL JSM-6610LV) and XPS (ESCALAB 250Xi XPS) approaches were used for surface analysis. The steel coupons were abraded mechanically with various grits of SiC paper and the dusts generated from the process were removed by washing in distilled water and ethanol. The dried samples were submerged for 24 h at 25 °C in 3.5% NaCl saturated with CO_2_ with and without the TBA. After 24 h immersion time, the specimens were removed from the environment, cleaned with distilled water and dried at room temperature before surface investigation. The acceleration voltage used in SEM analysis was 10 kV. The FTIR analysis was carried out on the adsorbed inhibitor film on the surface of specimen after immersion for 24 h at room temperature and also on the pure TBA for comparison purposes.

## Results and Discussion

### Variation of open circuit potential (OCP)

**Figure 2:**
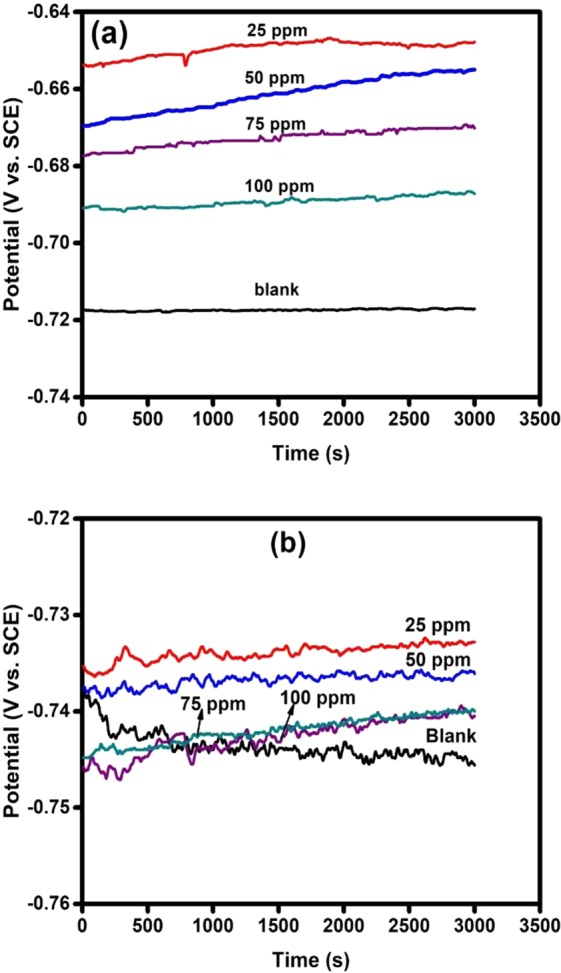
Variation of open circuit potential with time for API 5 L X60 mild steel in CO_2_ saturated 3.5% NaCl in the absence and presence TBA at (**a**) pH 4 and (**b**) pH 6.

It is an essential requirement that a steady state condition be established before the commencement of electrochemical impedance measurements. Practically, this is impossible since corrosion process is dynamic in nature. However, reliable electrochemical results can still be obtained if a pseudo steady condition is attained. For the purpose of fulfilling this important requirement, the working electrode was immersed in test solutions for approximately 3000 s. Figure 2 displays the variation of open circuit potential with time domain for API 5 L X60 steel in 3.5% NaCl CO_2_-saturated solution devoid of and containing various concentrations of TBA at (a) pH 4 and (b) pH 6. As could be seen in the figure, the allowed time was sufficient for a pseudo steady state condition to be established. It is however observed from the figure that the open circuit potential was affected by the composition of the solution and pH. Generally, a nobler OCP was recorded in pH 4 solution (Fig. 2(a)) than pH 6 solution (Fig. 2(b)). Pourbaix^22^ had predicted that solution pH affects the dissolution behavior of iron and its alloys by altering the stability of adsorbed film. In Fig. 2(a) (pH 4), the presence of TBA display OCP towards anodic direction relative to that of the blank. This suggests that TBA in the studied system exerted greater inhibiting effect on anodic reactions than cathodic reactions^23^. A precise categorization of an inhibitor as either anodic, cathodic, or mixed type requires an OCP displacement of up to 85 mV with respect to that of blank solution^24^. In Fig. 2(b) (pH 6), the lower and higher concentrations are seen to behave differently. For instance, 25 ppm and 50 ppm shifted OCP to nobler values while 75 ppm and 100 ppm initially displaced OCP towards more negative values relative to the OCP of the blank. Similar observation has been documented in the corrosion literature^25,26^ and was associated with adsorption or desorption of adsorbed films from substrate surface.

### Effect of inhibitor concentration

**Figure 3:**
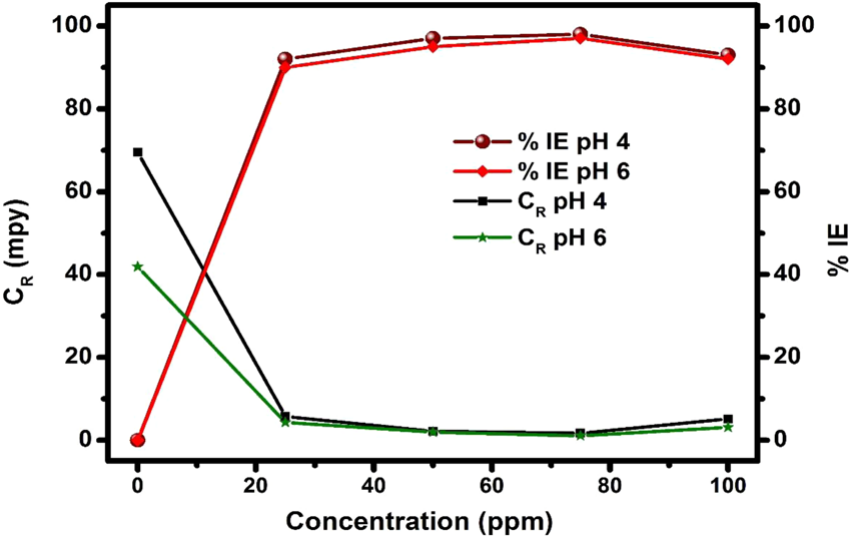
Variation of corrosion rate and inhibition efficiency against concentration for API 5 L X60 mild steel in CO_2_ saturated 3.5% NaCl in the absence and presence TBA at pH 4 and 6 from LPR measurements.

The effect of concentration on the TBA corrosion inhibition performance for API 5 L X60 steel in 3.5% NaCl CO_2_-saturated solution was examined using LPR approach at 25 °C at pH 4 and 6. Figure 3 displays the variations C_R_ (corrosion rate) and %IE with TBA concentration for the tested environment acidified to constant pH levels. Three observations can be highlighted. First, the corrosion rate in the blank solution at pH 4 (69.9 mpy) is notably higher than that at pH 6 (41.3 mpy). The higher corrosion rate in lower pH solution has been attributed to the dominating cathodic reaction^22^. Hydrogen reduction is the dominant cathodic reaction at pH 4 while reduction of carbonates is the predominant at pH 6^27–29^. Second, the overall performance of the inhibitor appears to be the same for both pH environments. For example, the corrosion rate dropped sharply on introduction of 25 ppm of TBA and inhibition efficiencies of 91% and 90% for pH 4 and 6, respectively are observed. Third, increasing TBA concentration from 25 to 75 is accompanied with slight improvement in the %IE at both pH levels. An increment in TBA amount up to 100 ppm resulted in a slight decline in %IE. This kind of behavior has been reported by some researchers^30,31^ and was attributed to the dependence of adsorption coverage on the inhibitor concentration. At concentrations below a critical value, the %IE increases with increased inhibitor concentration due to greater adsorption coverage. As the inhibitor concentration is raised above the critical value, adsorption coverage reduces owing to increased lateral repulsion between the inhibitor molecules.

**Figure 4:**
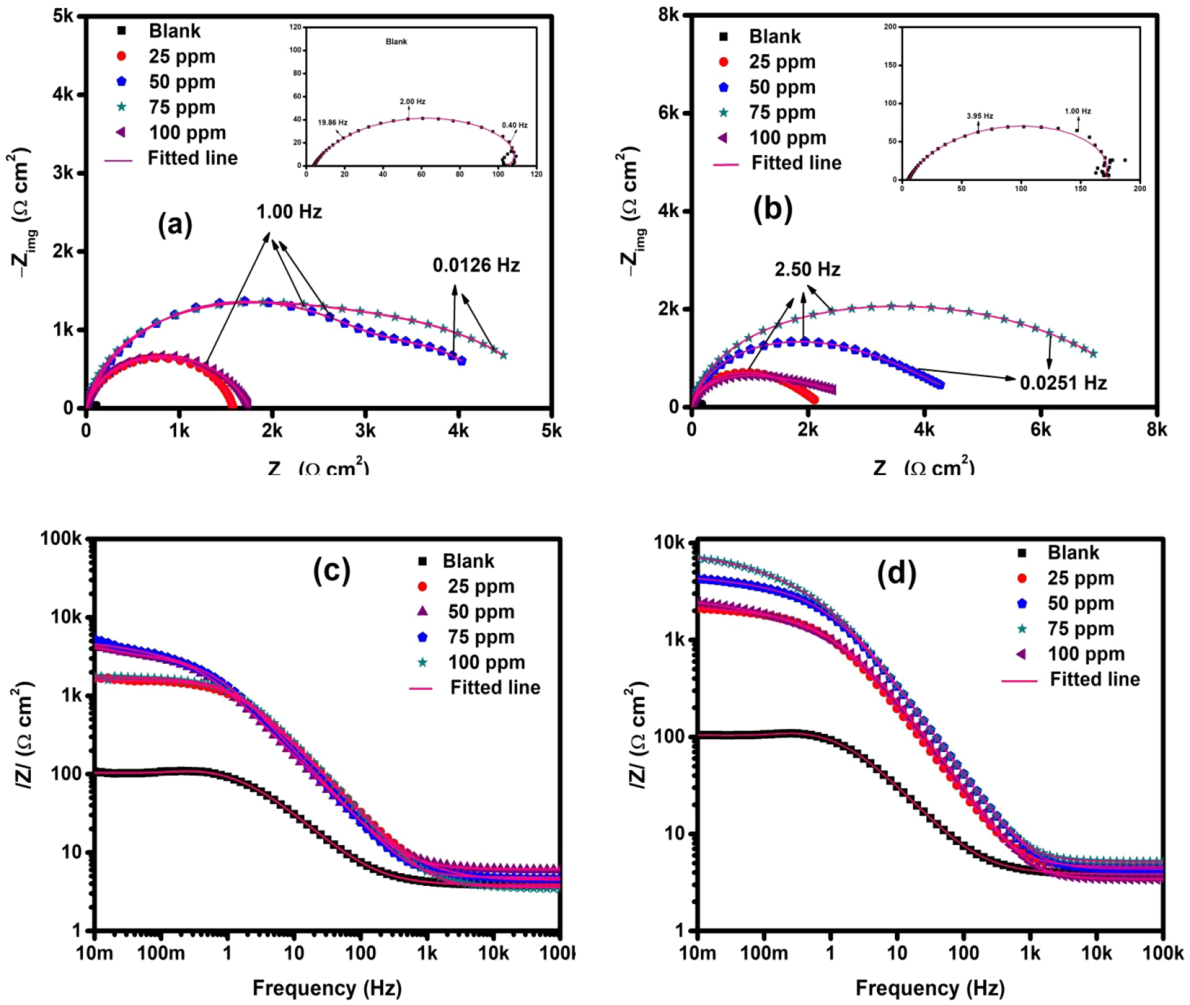
EIS Spectra for the behavior of API 5 L X60 mild steel in 3.5% CO_2_ saturated NaCl with and without TBA at 25 °C (**a**) Nyquist plot; pH = 4 (**b**) Nyquist plot; pH = 6 (**c**) Bode plot; pH = 4 and (**d**) Bode plot; pH = 6.

EIS approach was employed to further probe the performance of TBA in the considered aggressive medium. The impedance spectra for API 5 L X60 steel in blank solution and in TBA containing solutions (25–100 ppm) at two pH levels of 4 and 6 are shown in Fig. 4(a,b). TBA introduction has clearly changed the behavior of the steel and this can be visualized in two ways: First, the diameter of the semicircle which is related to R_ct_ (charge transfer resistance) increased with TBA addition and its magnitude increased with increasing TBA quantity up to 75 ppm. This reflects an improvement in the resistive property of the substrate against corrosion. At 100 ppm, the charge transfer resistance decreased indicating a drop in %IE compared to 50 and 75 ppm concentrations. Second, the inductive loop noted in the blank solution at low frequencies did not appear in TBA containing solutions. This is believed to be the result of active dissolution of the metal followed by adsorption of intermediate products (FeOH_ads_) in CO_2_ environment^32–37^. This illustrates the direct inhibitor effect on the dissolution process of the steel in CO_2_ corrosion. The formation of FeOH_ads_ as an intermediate during the dissolution of steel has long been reported in acidic media^38^ and in CO_2_ saturated environment^32–37^. The mechanism of intermediate product formation has been described by the following reactions^39^:

4 $${\rm{Fe}}+{{\rm{H}}}_{{\rm{2}}}{\rm{O}}\leftrightarrow {{\rm{FeOH}}}_{{\rm{adsorbed}}}+{{\rm{H}}}^{+}+{{\rm{e}}}^{-}$$

5 $${{\rm{FeOH}}}_{{\rm{adsorbed}}}\to {{{\rm{FeOH}}}^{+}}_{({\rm{sol}})+}+{{\rm{e}}}^{-}$$

6 $${{{\rm{FeOH}}}^{+}}_{({\rm{sol}})}+{{\rm{H}}}^{+}\leftrightarrow {{\rm{Fe}}}^{++}+{{\rm{H}}}_{{\rm{2}}}{\rm{O}}$$

**Figure 5:**
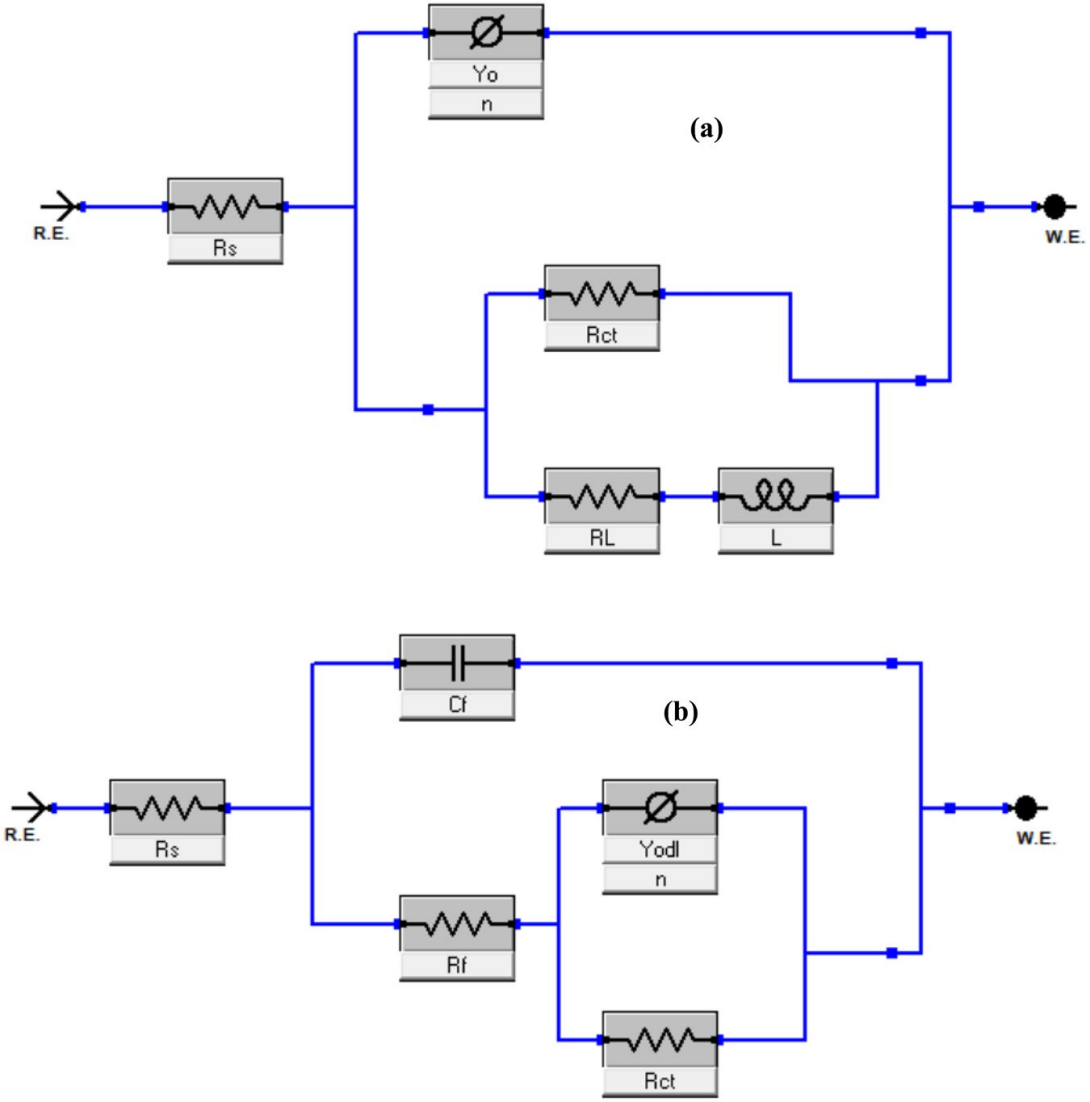
Equivalent circuit diagram used to fit impedance data (**a**) Blank and (**b**) presence of inhibitor.

Figures 4(c,d) show Bode plots for pH 4 and 6 CO_2_ environment, respectively. It is seen that the resistive impedance at the low frequency regime increase and reach its peak at the optimum TBA amount of 75 ppm and dropped thereafter. The ECs (equivalent circuits) deployed for the fitting of the EIS graphs which are similar to the ones earlier reported in Usman *et al*.^16^ are presented in Fig. 5 and the obtained parameters are listed in Table 2. The goodness of fit is given in terms of χ^2^ (chi squared values). In the ECs, R_s_ = solution resistance, R_ct_ = charge transfer resistance, R_f_ = film resistance, CPE_dl_ = di-layer capacitance represented as a constant phase element, C_f_ = film capacitance, R_L_ = inductive resistance, and L = inductance. The CPE describes non-ideal Faradaic capacitor and its impedance is given by:

7 $${Z}_{CPE}={Y}_{o}^{-1}{(jw)}^{-n}$$

**Table 2:** Impedance parameters for API X60 steel in CO_2_ saturated 3.5% NaCl without and with different concentrations of TBA at 25 °C at pH 4 and 6.

Conc.(ppm)	R_s_ (Ω cm^2^)	R_ct_ (Ω cm^2^)	R_f_ (Ω cm^2^)	Y_0_ × 10^4^ (s^n^/Ω cm^2^)	n	C_f_ (µF/cm^2^)	χ^2^ × 10^4^	%IE
**pH 4**								
Blank	4.9 ± 0.03	135.4 ± 0.33	—	7.91 ± 0.00	0.79 ± 0.01	−	5.4	−
25	4.5 ± 0.05	1580.0 ± 0.14	2.8 ± 0.06	0.94 ± 0.00	0.83 ± 0.00	19	5.0	91.2
50	4.6 ± 0.05	3762.0± 0.90	6.2 ± 2.99	1.40± 0.00	0.78± 0.03	38	9.0	96.3
75	4.9± 0.04	4748.0 ± 4.17	8.8 ± 1.73	1.34± 0.00	0.64± 0.00	60	4.0	97.1
100	3.6± 0.03	1696.0± 1.76	3.1 ± 1.52	0.75± 0.00	0.81± 0.04	29	8.3	91.8
**pH 6**								
Blank	5.3 ± 0.03	166.7 ± 1.30	—	5.20 ± 0.00	0.78± 0.03	—	2.3	—
25	4.6 ± 0.04	2100.0 ± 3.58	5.9 ± 2.66	1.31 ± 0.00	0.78± 0.00	48	1.8	91.8
50	4.5 ± 0.04	4342.0 ± 9.19	9.3 ± 6.17	0.94 ± 0.00	0.77± 0.00	33	1.9	96.0
75	5.3 ± 0.04	7875.0 ± 7.24	9.9 ± 3.61	1.05 ± 0.00	0.61± 0.00	35	2.6	97.8
100	3.5 ± 0.03	2716.0 ± 5.11	4.4 ± 2.46	2.32 ± 0.00	0.81± 0.00	48	1.8	93.7

The CPE represents an ideal faradaic capacitor as n approaches 1 (Y_o_ = C) and simplifies to a pure resistor as n approaches 0 (Y_o_ = 1/R). Table 2 reveals that the solution resistance (R_s_) is not varying significantly with the addition of the inhibitor for both solutions. However, the charge transfer resistance (R_ct_) changed dramatically with the introduction of 25 ppm of TBA and increased appreciably with increased inhibitor amount up to 75 ppm. Increase in the quantity of inhibitor to 100 ppm was accompanied with a noticeable drop in the R_ct_. The film resistance (R_f_) also increased with increase in the quantity of TBA up to 75 ppm and consequently decline as the concentration was raised to 100 ppm for pH 4 and 6 solutions. The double layer non-ideal capacitances of the electrode in the inhibitor-fortified solutions (Y_o_) are appreciably lower than in the free solution and showed no significant variation with the inhibitor concentration. Both increased R_ct_ and decreased Y_o_ in the inhibited solution signify a mechanism of suppression of the dissolution process probably because of adsorption of TBA molecules as compared to a monolayer water adsorption. The drop in the non-ideal capacitance in the inhibited solution could also be due to lower dielectric constant of the organic molecules as compared to water^32^. The %IE increased with increased concentration and reached the maximum values of 97.1 and 97.8% at 75 ppm concentration at pH 4 and 6, respectively. By increasing the concentration further, %IE decreased which is consistent with LPR results (Fig. 3). It is exciting to note that the double layer exponent n corresponding to the highest %IE in both solutions are the minimum values (0.64 and 0.61 for pH 4 and 6 solutions, respectively). It should be noted that the limiting value of n is 0.5 where the reactive species are under diffusion control. Similar behavior can be observed in the data reported by Espinoza-Vázquez *et al*.^31^ for perezone inhibition of 1018 steel immersed in CO_2_ saturated 3% NaCl solution. The minimum values of n could point to the development of full coverage of adsorbed TBA molecules on the steel surface resulting in diffusion controlled electrochemical reactions.

**Figure 6:**
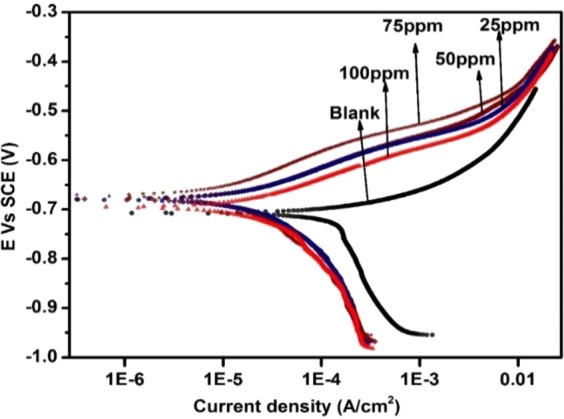
Potentiodynamic polarization curves for X60 mild steel in 3.5% CO_2_ saturated NaCl without and with 50 ppm TBA at pH 4, 25 °C and 2 h immersion.

Figure 6 presents typical polarization curves for API 5 L X60 steel in CO_2_-saturated 3.5% NaCl solution devoid of and containing TBA at 25 °C and pH of 4. The electrochemical parameters derived from the analysis of these curves are displayed in Table 3. As clearly seen in the table, the addition of TBA to the corrosive medium resulted in equal reduction of the cathodic current densities for all inhibitor concentrations when compared at the same potential. However, the anodic current density decreased proportionally with the concentration reaching the lowest value in the solution containing 75 ppm TBA. A further increase in TBA concentration to 100 ppm was accompanied with an increase in the anodic current density over the whole anodic region. Table 3 also reveals that the addition of TBA resulted in a shift in E_corr_ values towards more noble values. Moreover, i_corr_ decreased with addition of TBA reaching the lowest value of 2.69 µA/cm^2^ at 75 ppm concentration with the highest %IE of 98.9. The corrosion current density increased to 9.62 µA/cm^2^ as the TBA concentration was raised to 100 ppm resulting in a slight drop in %IE. Both the anodic and the cathodic Tafel (β_a_ & β_c_) constants have changed in the presence of the inhibitor. This suggests that TBA functioned as a mixed type inhibitor. The mechanism of inhibition of the cathodic reaction seems to be concentration-independent whereas the influence of TBA on the anodic reaction appears to be sensitive to the range of concentration examined (25–100 ppm).

**Table 3:** Tafel polarization parameters for API X60 steel in CO_2_ saturated 3.5% NaCl in the absence and presence of different concentrations of TBA at pH 4.

Concentration (ppm)	E_corr_ (mV/SCE)	i_corr_ (µA/cm^2^)	β_a_ (mV/decade)	-β_c_ (mV/decade)	%IE
Blank	−707	231	55.5	30.6	−
25	−680	7.42	37.7	46.4	96.8
50	−680	6.23	32.2	38.4	97.3
75	−672	2.69	26.5	26.6	98.9
100	−696	9.62	23.8	23.7	95.8

### Effect of immersion time

The effect of exposure duration on the corrosion inhibition of TBA was evaluated using LPR method at a constant inhibitor concertation of 50 ppm in a solution of fixed pH of 4 at 25 °C. The corrosion rates and the polarization resistances for the blank and the inhibited solution are presented in Table 4 for 2, 12, 24, 48, and 72 h of continuous immersion. As could be seen, the dissolution rate for the unfortified solution increased with immersion time while that of the fortified solution decreased slightly with time. Increases in corrosion rates for the blank sample and decreases in the corrosion rates for the inhibited solution with longer exposure period underlines the effectiveness of the inhibition process.

**Table 4:** Linear polarization resistance parameters for API X60 steel in CO_2_ saturated 3.5% NaCl without and with 50 ppm TBA at pH 4 after different immersion times.

Time (h)	Blank		50 ppm		%IE
	C_R_ (mpy)	R_p_ (Ω cm^2^)	C_R_ (mpy)	R_p_ (Ω cm^2^)	
2	69.5	142.6	2.1	4655	96.9
12	75.6	131.1	1.8	5469	97.6
24	81.5	121.7	1.6	6248	98.1
48	93.7	105.8	1.5	6701	98.4
72	95.8	103.4	1.4	7202	98.6

**Figure 7:**
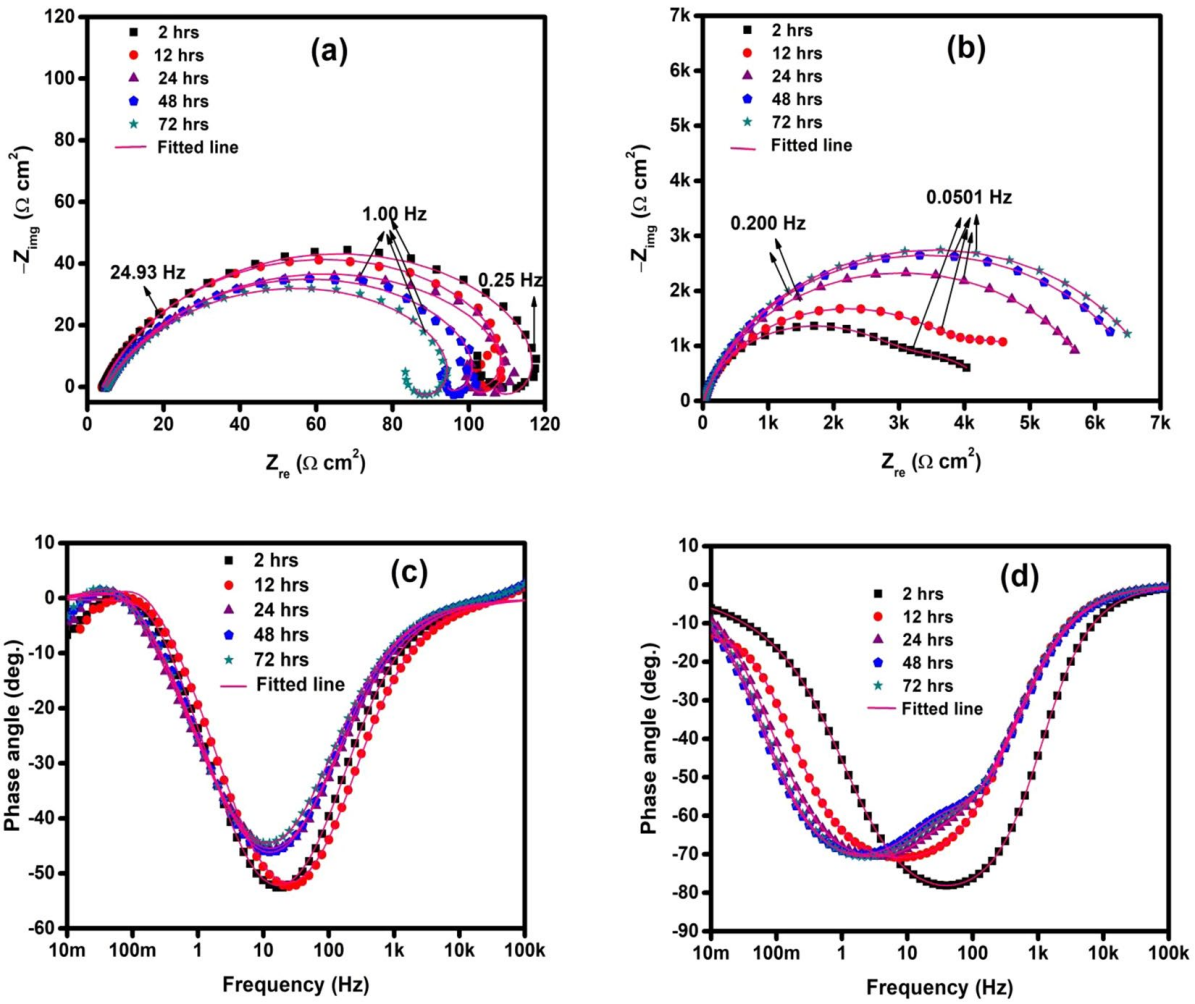
EIS plots for API 5 L X60 steel in CO_2_ saturated 3.5% NaCl with and without 50 ppm TBA at 25 °C and pH 4 (**a**) Nyquist for blank (**b**) Nyquist for 50 ppm TBA (**c**) phase angle for blank (**d**) phase angle for 50 ppm TBA at different immersion times.

The impedance plots in Nyquist and Bode phase angle representations for the studied steel in 3.5% NaCl solution saturated with CO_2_ at pH of 4 without and with 50 ppm TBA at different immersion times are depicted in Fig. 7. The Nyquist spectra for the blank solution (Fig. 7(a)) exhibits inductive character at low frequencies for all exposures duration due to adsorption of an intermediate product during the dissolution process^31–37^. This behavior is clearly illustrated in the phase angle vs. frequency plot in Fig. 7(c) which displays positive phase angles at low frequencies for all exposure times in the blank solution. At intermediate frequencies, it is evident that there is a single time constant for all exposures suggesting the absence of the formation of carbonate precipitation film. The phase angle is maximum for 2 and 12 h exposures and dropped for 24, 48, and 72 h and the maximum value shifted slightly to lower frequency. The decrease in the maximum phase angle might suggest a loss in the double layer capacitance at longer exposure times. Fig. 7(d) shows the phase angle vs. frequency plots in the inhibited solution for various exposure times. Single time constant is apparent in the plots for 2 and 12 h while the phase angle responses for 24, 48, and 72 h show the presence of diffuse double time constants with small difference in the intermediate frequency region. In addition, the maximum phase angles are more negative in the inhibitive solution than in the free solution which is related to the increase of the capacitance character due to the inhibitor film^40^. Moreover, the additional peak at medium frequencies during longer exposure times is indicative of the presence of an adsorbed inhibitor film on the substrate surface and this layer is responsible for improved protection performance. Furthermore, Fig. 7(d) shows that the maximum phase angle shifted to lower frequencies at longer immersion time. This could be attributed to enhanced film capacitance or film resistance or both according to the relationship between the angular frequency (ω_max_) and the double layer resistance R_dl_ and capacitance C_dl_ (ω_max_ = 1/(R_dl_*C_dl_)). The equivalent circuits shown in Fig. 5 were used to fit the experimental data in order to generate the electrochemical parameters. Table 5 presents EIS parameters for the influence of immersion time on the corrosion process of API 5 L X60 steel in CO_2_ environment at 25 °C and pH 4. The R_ct_ in the blank solution decreased with immersion time while the charge transfer resistance in TBA containing solution increased with time which indicates improved inhibition with immersion time. Table 5 also indicate that the film resistance (R_f_) and the film capacitance (C_f_) increased with longer exposure time. This might be related to thickening of the adsorbed inhibitor film with exposure time.

**Table 5:** EIS parameters for API X60 mild steel in CO_2_ saturated 3.5% NaCl without and with 50 ppm TBA at pH 4 at different immersion times.

Conc.	Time (h)	R_s_ (Ω cm^2^)	R_ct_ (Ω cm^2^)	R_f_ (Ω cm^2^)	Y_0×10_^4^ (s^n^/Ω cm^2^)	n	C_f_ (µF cm^−2^)	χ^2^ × 10^4^	%IE
Blank	2	4.9 ± 0.03	135.4 ± 0.33	—	7.91 ± 0.00	0.79 ± 0.01	—	5.4	—
	12	4.9 ± 0.06	125.3 ± 0.72	—	14.92 ± 0.00	0.76 ± 0.02	—	6.4	—
	24	4.9 ± 0.06	117.1 ± 0.73	—	16.2 ± 0.00	0.74 ± 0.02	—	4.5	—
	48	4.9 ± 0.06	107.7 ± 0.76	—	15.70 ± 0.00	0.72 ± 0.02	—	2.7	—
	72	5.0 ± 0.05	100.9 ± 0.43	—	15.17 ± 0.00	0.76 ± 0.01	—	2.8	—
50 ppm	2	4.6 ± 0.05	3762.0 ± 0.90	6.2 ± 2.99	1.40 ± 0.00	0.78 ± 0.03	38	9.0	96.3
	12	6.1 ± 0.05	4656.0 ± 6.86	8.6 ± 1.01	2.08 ± 0.00	0.77 ± 0.00	44.1	1.2	97.0
	24	6.2 ± 0.05	6225.0 ± 1.43	19.4 ± 1.66	2.41 ± 0.00	0.76 ± 0.00	53.7	8.0	98.0
	48	6.2 ± 0.05	7032.0 ± 1.07	26.4 ± 0.46	2.50 ± 0.00	0.79 ± 0.02	57.6	1.5	98.4
	72	6.2 ± 0.05	7243 ± 1.11	26.6 ± 1.76	2.82 ± 0.00	0.79 ± 0.00	57.7	1.2	98.5

### Influence of temperature

The corrosion characteristics of API 5 L X60 steel immersed in 3.5% NaCl solution saturated with CO_2_ without and with 50 ppm TBA was examined in the temperature range of 25 to 80 °C. Results of LPR measurements performed to assess the temperature factor on the dissolution characteristic of API 5 L X60 steel in the sweet corrosive medium with and without TBA at pH 4 are presented in Table 6. It is seen from the table that the corrosion rate in the blank solution increased at elevated temperatures which might be associated with the absence of carbonate protective scales^41^. The corrosion rates for the inhibited solution increased slightly with the solution temperature. However, the inhibition efficiency remained almost constant over the range of temperature investigated. The influence of temperature on the inhibition process is a complex phenomenon because of the numerous competing factors that affect the kinetics of inhibitor adsorption/desorption, electrochemical reactions, and diffusion of the reactive species. For example, rise in temperature may alter the chemical behavior of inhibitor molecules such that the electron densities at the centers of the molecules increase and improves molecular adsorption^42^. Also, the inhibitor itself may undergo decomposition and/or rearrangement^43^ and this could have serious influence on the inhibition efficiency. Therefore, the effect of temperature required further investigation.

**Table 6:** Linear polarization resistance parameters for API X60 mild steel in CO_2_ saturated 3.5% NaCl without and with 50 ppm TBA at pH 4 at different temperatures.

Temperature (°C)	Blank	50 ppm			
	R_p_ (Ω cm^2^)	C_R_ (mpy)	R_p_ (Ω cm^2^)	C_R_ (mpy)	%IE
25	142.6	69.5	4655	2.1	96.9
40	93.5	106.1	3952	2.5	97.6
60	64.5	153.7	3006	3.3	97.9
80	56.6	175.2	2801	3.5	98.0

**Figure 8:**
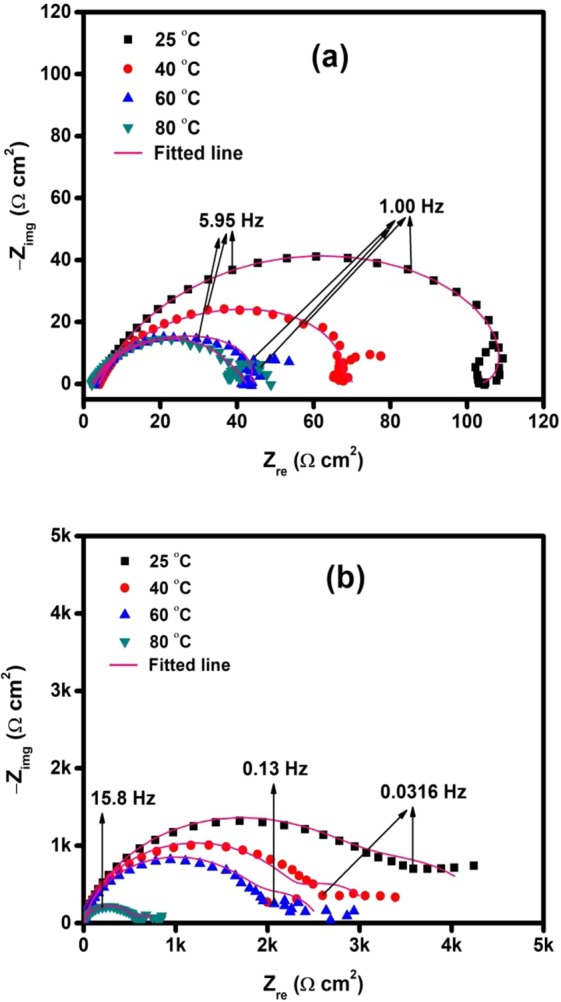
EIS Nyquist Spectra for the behavior of API 5 L X60 mild steel in 3.5% CO_2_ saturated NaCl (**a**) without and (**b**) with 50 ppm of TBA at different temperatures.

The influence of temperature on the adsorption behavior of TBA was also investigated using EIS and Fig. 8 presents the obtained Nyquist plots. The parameters associated with the plots are listed in Table 7. The data indicate that the charge transfer resistance (R_ct_) decreased with increase in temperature from 25 to 80 °C for the blank and the inhibited solutions. The %IE calculated using LPR data (Table 6) and EIS data (Table 7) are consistent in showing that the %IE slightly increased with temperature. Several researchers have attributed increased %IE with rise in temperature to the difference in E_a_ (activation energy) of corrosion processes in the uninhibited and inhibited solutions^43^. At a given concentration, if %IE varies inversely with temperature, the implication is that the E_a_ of metal dissolution in the presence of the inhibitor is higher than that of the uninhibited solution such behavior is often associated with prevailing physisorption. On the other hand, situations where %IE increases with temperature rise, then E_a_ in the inhibited system would be smaller than the uninhibited solution and such would imply chemisorption^43,44^.

**Table 7:** EIS parameters for API X60 steel in CO_2_ saturated 3.5% NaCl without and with 50 ppm TBA at pH 4 at different temperatures.

Conc.	Temp (°C)	R_s_ (Ω cm^2^)	R_ct_ (Ω cm^2^)	R_f_ (Ω cm^2^)	Y_0×10_^4^ (Ω.cm^2^ S^−n^)	n	C_f_ (µF/cm^2^)	χ^2^ × 10^–4^	%IE
Blank	25	4.9 ± 0.03	135.4 ± 0.33	—	7.91 ± 0.00	0.79 ± 0.01	—	5.4	—
	40	4.2 ± 0.04	65.3 ± 0.56	—	12.1 ± 0.00	0.73 ± 0.02	130	0.2	—
	60	2.8 ± 0.03	41.1 ± 0.42	—	13.1 ± 0.00	0.74 ± 0.02	130	0.39	—
	80	2.1 ± 0.03	40.4 ± 0.21	—	13.9 ± 0.00	0.77 ± 0.04	0.16	0.37	—
50 ppm	25	4.6 ± 0.05	3762.0 ± 0.90	6.2 ± 2.99	1.40 ± 0.00	0.78 ± 0.03	38	9.0	96.3
	40	5.0 ± 0.04	2697.0 ± 1.36	4.9 ± 0.38	1.00 ± 0.00	0.73 ± 0.00	0.44	4.0	97.4
	60	2.8 ± 0.02	2228.0 ± 0.09	2.7 ± 0.03	0.73 ± 0.00	0.74 ± 0.00	0.22	4.5	98.0
	80	2.3 ± 0.02	2190.0 ± 0.60	2.4 ± 0.13	1.97 ± 0.00	0.77 ± 0.00	0.50	5.6	98.2

**Figure 9:**
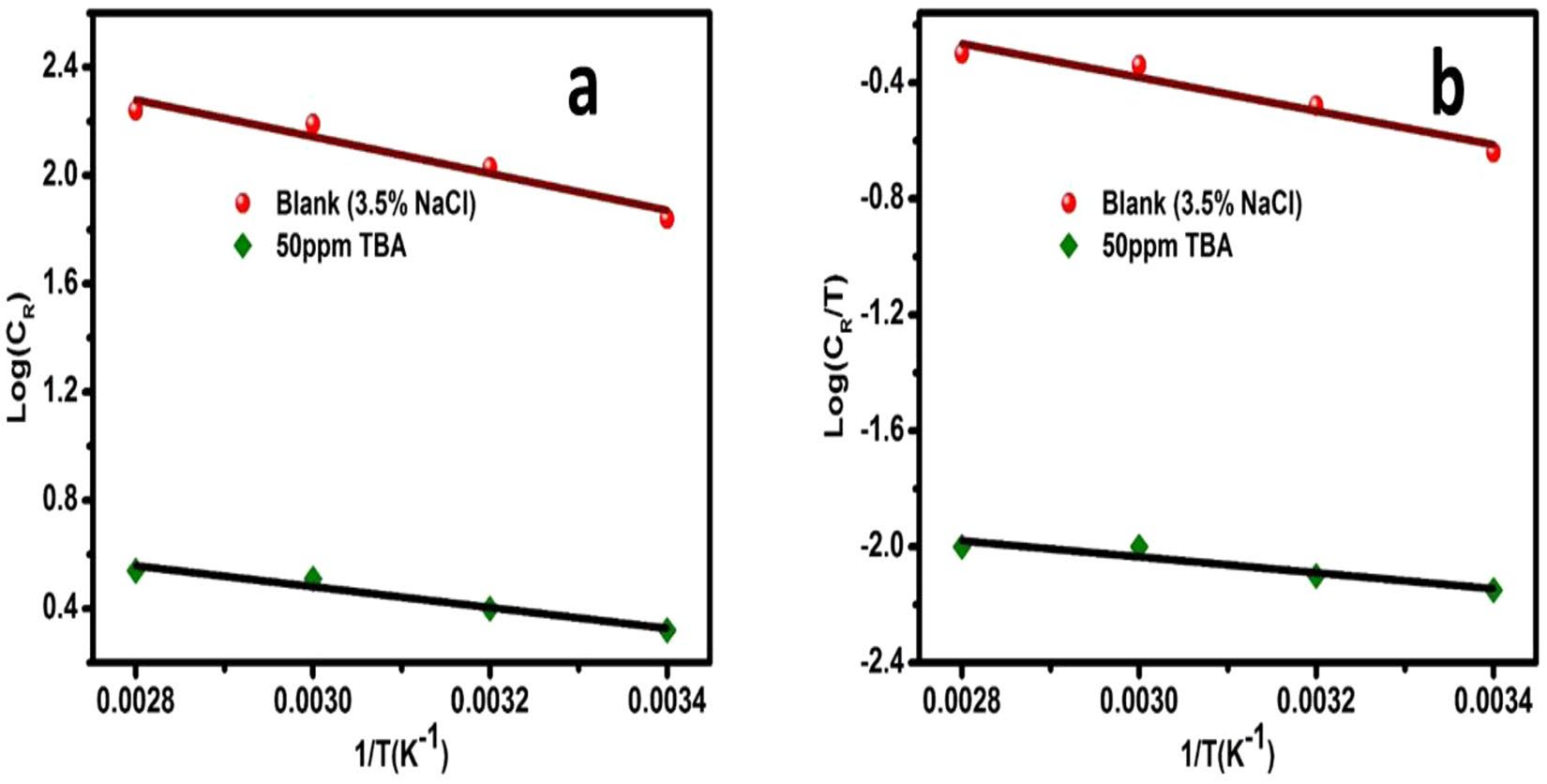
(**a**) Arrhenius and (**b**) Transition state plots for API 5 L X60 steel in 3.5% NaCl in the absence and presence of 50 ppm TBA.

The E_a_ for the corrosion process can be calculated from Arrhenius-type relationship which relates the variation in the corrosion rate to the temperature (Equation 8)^43–45^:

8 $${C}_{R}=A\,exp(\frac{-{E}_{a}}{RT})$$

where C_R_ = the corrosion rate, R = molar gas constant, A = the Arrhenius pre-exponential factor, and T is the absolute temperature. A plot of C_R_ as a function of the inverse of temperature in Kelvin (Fig. 9(a)) gives −E_a_/2.303 R as the slope from which the value of E_a_ was extracted. The calculated E_a_ values are listed in Table 8. The apparent E_a_ of corrosion in the inhibited solution is 46% lower than that of the uninhibited system. This may mean that coordinate type of bonding has taken place between the lone pairs of electrons of the inhibitor and the partially filled d orbitals of Fe atoms at the surface. Several researchers have reported lower E_a_ in the presence of the inhibitor compared to the blank solution and was associated with chemisorption^42–44^.

**Table 8:** Activation parameters for API X60 steel in 3.5% NaCl without and with 50 ppm TBA at pH 4.

Concentration (ppm)	E_a_ (kJ mol^−1^)	ΔH_a_ (kJ mol^−1^)	ΔS_a_ (J K^−1^ mol^−1^)
Blank	13.6	11.1	−171
50	7.4	5.3	−221

To obtain the ΔH_a_ (enthalpy of activation) and the ΔS_a_ (entropy of activation), an alternative formulation of the Arrhenius equation is used^42^:

9 $${i}_{corr}=\frac{RT}{Nh}exp(\frac{\Delta {S}_{a}\,}{R})exp(-\frac{\Delta {H}_{a}\,}{RT})$$

where N = Avogadro’s number, h = Planck’s constant. Fig. 9(b) shows a plot of log (i_corr_/T) vs. 1/T for the blank and inhibited solution. The slope and the intercept are −ΔH_a_/R*log e and log e* {log (R/Nh) + ΔS_a_/R} respectively from which ΔH_a_ and ΔS_a_ were estimated and given in Table 8. The obtained ΔH_a_ value is positive in both systems and is an indication that the corrosion process of the metal in both inhibited and uninhibited environments is an endothermic and suggests a chemisorption interaction between the inhibitor and the metal surface^43^. The negative values for ΔS_a_ in both environments suggest that the activated complex in the rate determining step represents an association rather than a dissociation step^43^. This can be rationalized as a decrease in disorder (increase in order) as the reaction proceeds from reactants to the activated complex^43^.

### Adsorption isotherm study

It is widely reported that organic inhibitors adsorb on substrate surface to protect against corrosion^43,44^. This adsorption process is a quasi-replacement of adsorbed H_2_O molecules (nH_2_O _(ads)_) by inhibitor molecules in aqueous solution (Inh _(sol)_)^43,46^:

10 $${{\rm{Inh}}}_{(\mathrm{sol})}+{{\rm{nH}}}_{{\rm{2}}}{{\rm{O}}}_{(\mathrm{ads})}\to {{\rm{Inh}}}_{(\mathrm{ads})}+{{\rm{nH}}}_{{\rm{2}}}{{\rm{O}}}_{(\mathrm{sol})}$$

The degree of surface coverage of an inhibitor on a metal’s surface (θ) has been widely used to elucidate the mechanism of adsorption. The surface coverage (θ) can be shown to be directly related to %IE as follows (for effective inhibition system where the maximum inhibited corrosion rate is much less than the corrosion rate in the blank solution)^43^:

11 $${\rm{\theta }}= \% \mathrm{IE}/\mathrm{100}$$

The values of θ (obtained from LPR measurements) were fitted into different adsorption isotherm models in order to select the most suitable adsorption isotherm that explains the interaction between TBA and the metal surface. Langmuir adsorption isotherm was found to give the best and this isotherm is given as^47^:

12 $${\rm{\theta }}/({\rm{1}}\,-\,{\rm{\theta }})={{\rm{K}}}_{{\rm{ads}}}{{\rm{C}}}_{{\rm{inh}}}$$

where C_inh_ = TBA concentration in mol/L and K_ads_ = the equilibrium constant of adsorption. Equation (12) can be simplified and presented as^43^:

13 $${{\rm{C}}}_{{\rm{inh}}}/{\rm{\theta }}={1/K}_{{\rm{ads}}}+{{\rm{C}}}_{{\rm{inh}}}$$

**Figure 10:**
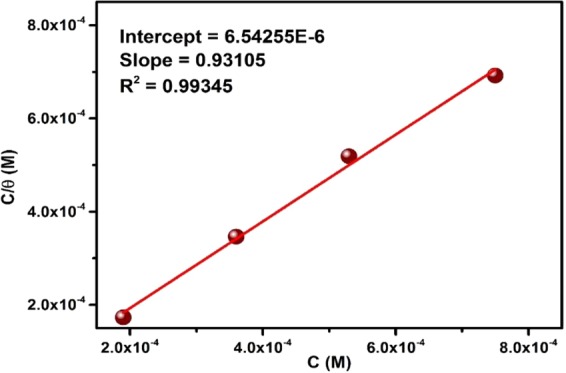
Langmuir adsorption isotherm of API 5 L X60 mild steel in 3.5% NaCl saturated with CO_2_ containing TBA at 25 °C and pH 4.

The variation of C_inh_/θ vs. C_inh_ for CO_2_-saturated 3.5% NaCl solution with 50 ppm TBA at pH 4 is depicted in Fig. 10. The curve is linear indicating that Langmuir adsorption isotherm is obeyed. The value of K_ads_ was estimated from the intercept to be 1.53 × 10^5^ mol/L. The ΔG°_ads_ (standard free energy of adsorption) is related to K_ads_ as follows:

14 $${{\rm{\Delta }}{\rm{G}}^\circ }_{{\rm{ads}}}=-\,{\rm{RT}}\,\mathrm{ln}\,(\mathrm{55}{\rm{.5}}\,{{\rm{K}}}_{{\rm{ads}}})$$

where T and R retain the meanings as defined in Eq. 8; 55.5 is the concentration of water in mol/dm^3^.

The value of ΔG°_ads_ calculated for TBA adsorption on API 5 L X60 steel in CO_2_ environment is estimated to be −40 kJ/mol which is in the range of values reported in the corrosion literature for chemical adsorption^48^. Hence, in this work it is plausible to say that TBA adsorbs on API 5 L X60 steel surface by chemisorption mechanism involving sharing of electrons or transfer of electrons from TBA molecules to the partially filled d-orbital of Fe atoms at the surface. The bigger value of K_ads_ and the negative value of ΔG°_ads_ indicate a strong interaction between TBA molecules and API 5 L X60 steel surface.

### Surface analyses

**Figure 11:**
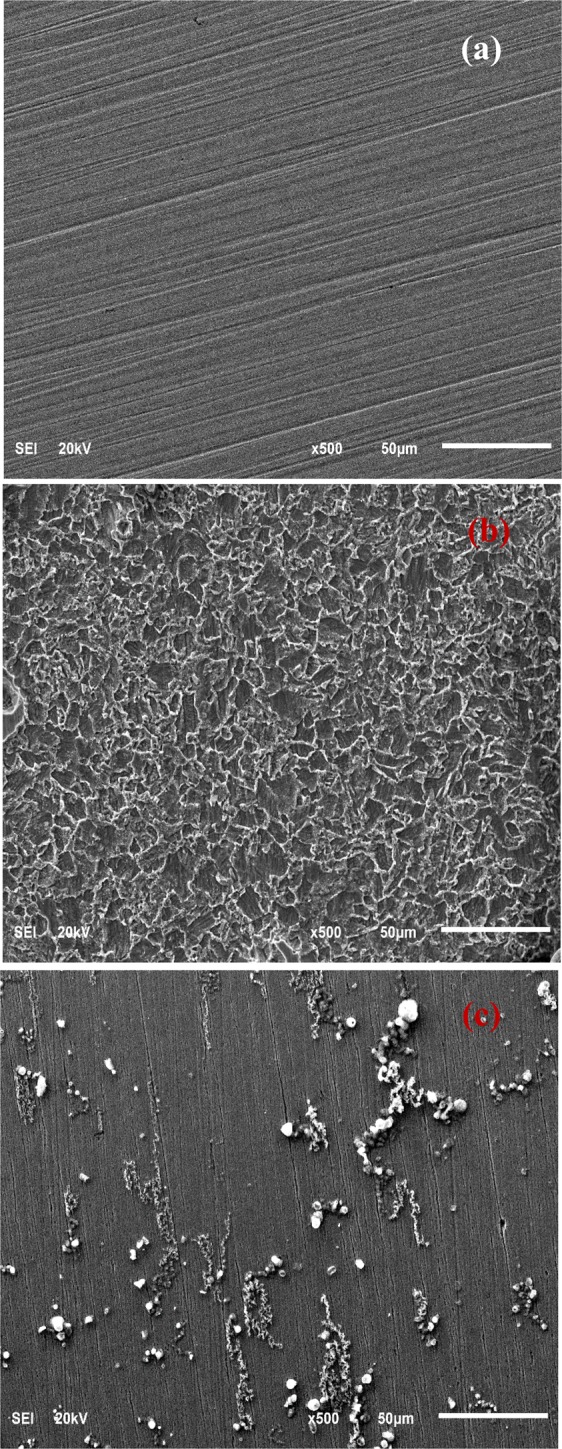
SEM images for API 5 L X60 mild steel in (**a**) before immersion, (**b**) after immerstion in CO_2_ –saturated 3.5% NaCl and (**c**) immersion in CO_2_ –saturated 3.5% NaCl with 50 ppm TBA.

The SEM pictures in Fig. 11 depict the samples before and after immersion in 3.5% NaCl saturated with CO_2_ in the absence and presence of 50 ppm TBA at 25 °C and pH 4. The smooth morphology of API 5 L X60 steel surface after abrasion is quite obvious in Fig. 11(a). After 24 h of immersion in the inhibitor-free solution, a severely damaged and coarse morphology due to the rapid and aggressive corrosion attack of API 5 L X60 steel in studied environment is observed (Fig. 11(b)). The corrosive attack was minimized in the system containing 50 ppm TBA (Fig. 11c); the steel surface remains relatively un-attacked probably due to the formation of adsorbed TBA film which protected the metal from corrosion. This supports the excellent inhibition process by TBA as was shown by the experimental results (Tables 1–3).

**Figure 12:**
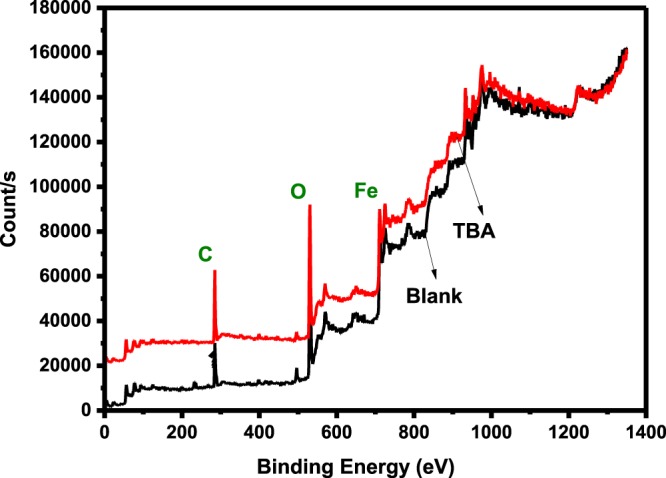
Survey scan of XPS spectra for API 5 L X60 mild steel surface product obtained after 24 h immersion in 3.5% NaCl at 25 °C and pH 4 without and with 50 ppm TBA.

**Figure 13:**
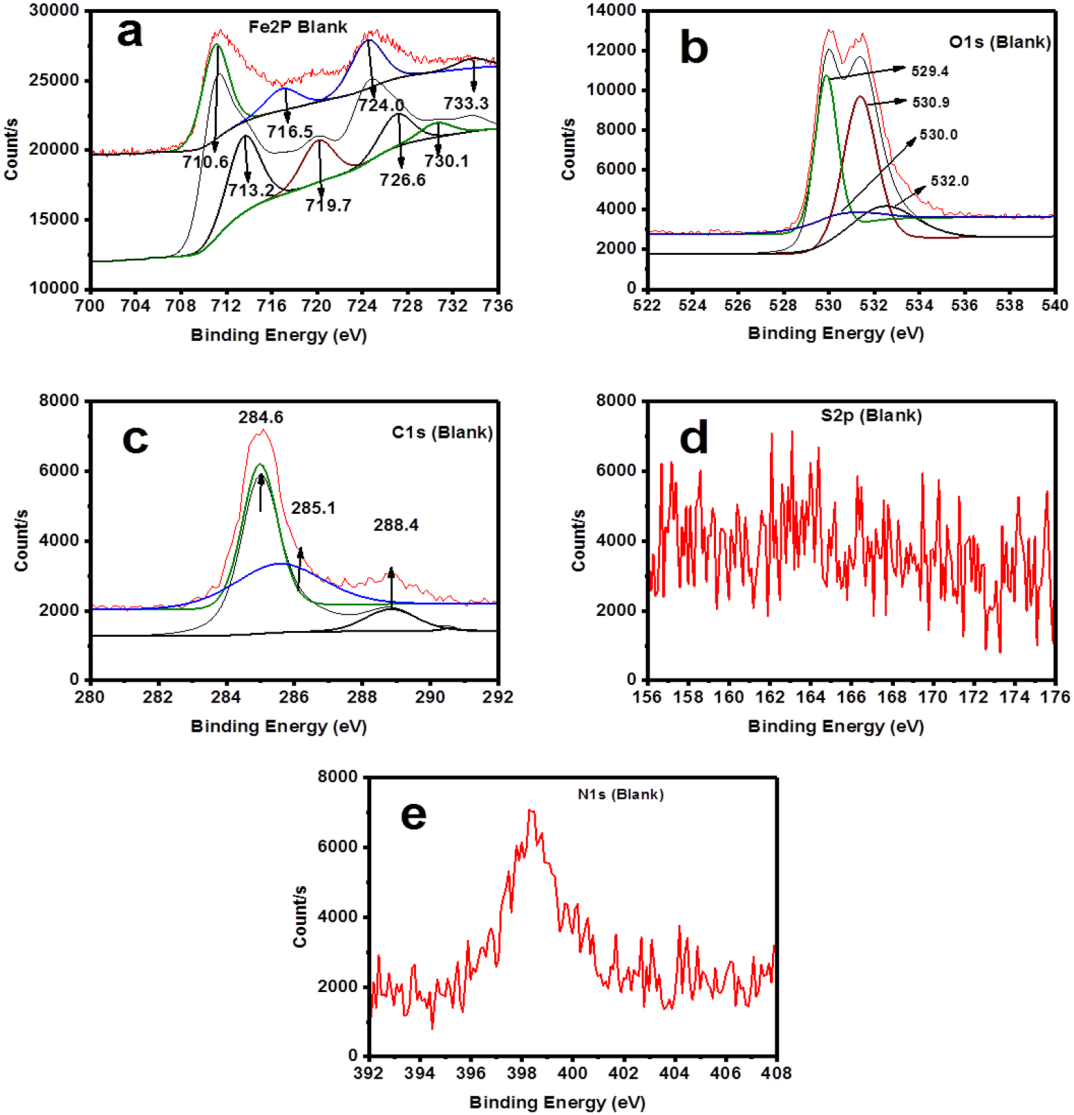
High Resolution XPS Spectra for API 5 L X60 mild steel after 24 h immersion at 25 °C and pH 4 in CO_2_ - saturated 3.5% NaCl: (**a**) Fe2p (**b**) O1s (**c**) C1s (**d**) S2p and (**e**) N1s.

**Figure 14:**
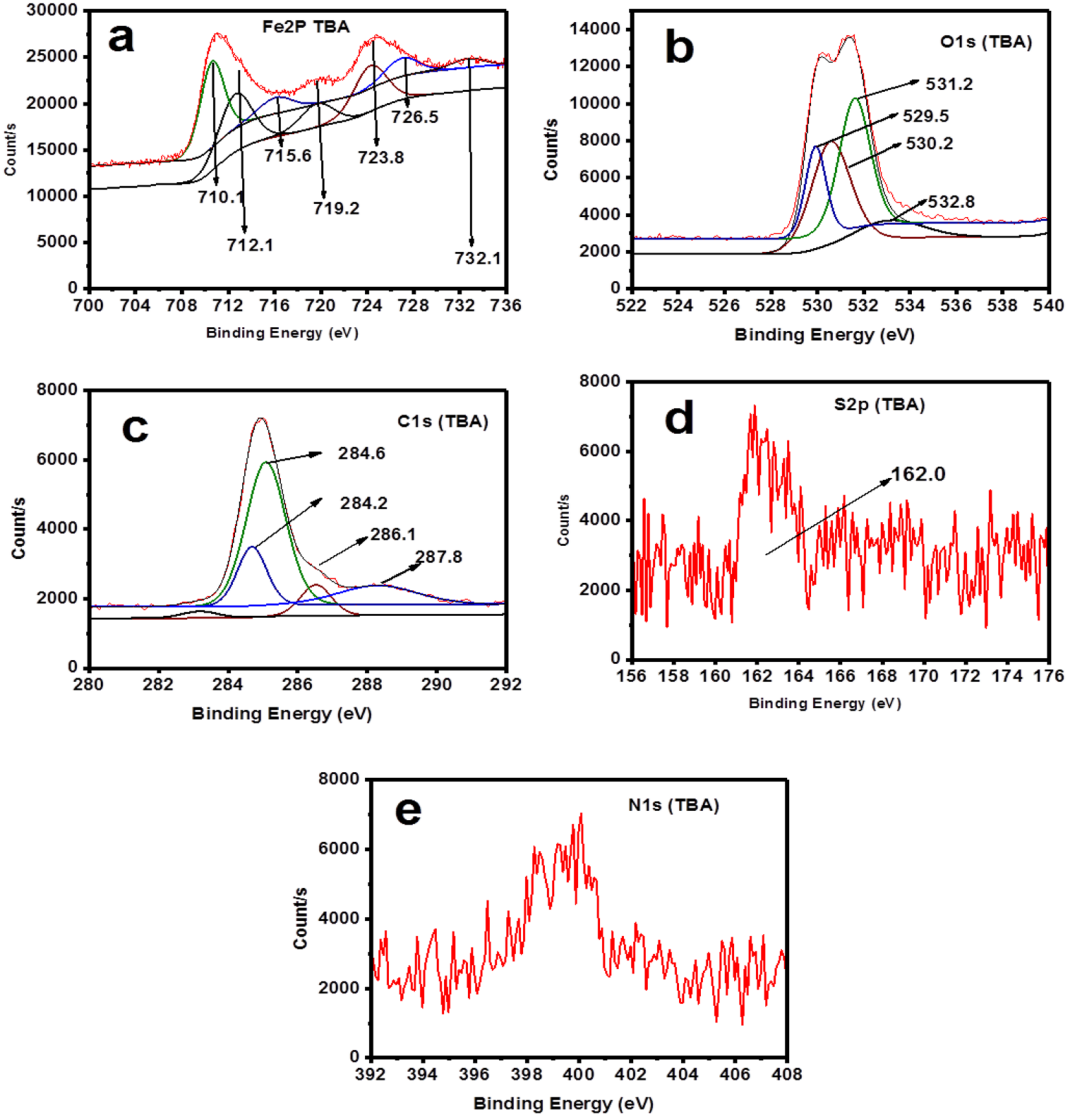
High Resolution XPS Spectra for API 5 L X60 mild steel after 24 h immersion at 25 °C and pH 4 in CO_2_ - saturated 3.5% NaCl with 50 ppm TBA: (**a**) Fe2p (**b**) O1s (**c**) C1s (**d**) S2p and (**e**) N1s.

XPS analysis for both wide and narrow scans were undertaken in the absence and presence of 50 ppm TBA at 25 °C and pH 4. Primary peaks detected in the wide scan for both the inhibited and blank specimens are C1s, O1s and Fe2p as could be seen in Fig. 12. The bands for O and C show prominence in the specimen immersed in solution inhibited with 50 ppm TBA compared to the blank. These elements are the basic component of TBA structure and the observed prominence is clear evidence of TBA adsorption. In the narrow scan for the blank (Fig. 13) and inhibited (Fig. 14), high resolution XPS analysis shows the absence of S in the blank sample but present in the TBA containing solution sample at 162 eV. This binding energy can be attributed to Fe-S interaction^49^. It is therefore obvious that TBA molecules adsorbed on the metal surface and this adsorption leads to the blockage of active sites on the metal surface thereby reducing corrosion. For the charge effects, the binding energies were amended by taking C1s to the band at 284.6 eV.

The standard binding energy for the formation of FeCO_3_ are C1s band at 289.4 eV, O1s peak at 531.9 eV, and Fe2p_3/2_ peak at 710.2 eV^16,49^. This is why according to Table 9, the peaks close to these values appeared in the uninhibited specimen indicating the presence of unstable carbonate layer formation. Furthermore, in Table 9 is listed the binding energy of the XPS bands, proportions (in atomic weight %), and their corresponding possible assignments.

**Table 9:** XPS spectral analysis for the surface products on API X60 steel after 24 h immersion in CO_2_ -saturated 3.5% NaCl in the absence and presence of 50 ppm TBA at pH 4.

Concentration (ppm)	Peak	Energy (eV)	Atomic Weight (%)	Possible Assignment	References
Blank	Fe2p	710.6	28.84	FeO, FeCO_3,_ Fe_2_O_3,_ FeCl_2_	^50^
		713.2	18.92	Fe_2_O_3_, FeOOH	^50^
		724.0	18.26	Fe_3_O_4,_ Fe_2_O_3_	^16,51^
	O1s	529.4	30.29	Fe_2_O_3_, FeO	^49^
		530.0	7.74	Fe_2_O_3_, FeO	^49,50^
		530.9	42.97	FeOOH,	^52^
		531.9	18.99	C=O	^16,50^
	C1s	284.6	50.16	C-C	^51^
		285.1	37.08	C-O-C	^49,53^
		288.4	12.77	-CO_3_	^49,51^
50	Fe2p	710.1	26.89	FeO_,_ Fe_2_O_3,_ FeCl_2_	^52^
		712.1	24.73	FeS, C=S_,_ FeOOH,	^54^
		723.8	19.75	Fe_3_O_4,_ Fe_2_O_3_	^51^
	O1s	529.5	16.91	Fe_2_O_3_, FeO	^16,49^
		530.2	35.95	Fe_2_O_3_, FeO	^52^
		531.2	36.97	FeOOH	^49,55^
		532.8	10.17	C=O	^53^
	C1s	284.2	9.50	C=C	^56^
		284.6	70.73	C-C	^51^
		286.0	3.56	C=N	^53^
		287.8	6.57	C=O, C-N	^16,51^
		287.9	9.65	C=O, C-N	^57^
	S2p	167.6	34.82	C=S, SO_2_	^58^
		162.0	65.18	FeS	^49,58^

## Conclusions

The conclusions drawn on the basis of the results obtained in this work are as follow:

1. TBA is effective in retarding CO_2_ corrosion of API 5 L X60 steel in the temperature range of 25–80 °C and pH 4 and 6.
2. The protection performance of TBA is a function of TBA concentration and the optimum concentration is 75 ppm at both pH 4 and 6. TBA exhibits excellent inhibition effect for up to 72 h of exposure time.
3. TBA behaves as a typical mixed type corrosion inhibitor.
4. The adsorption of TBA can be explained with Langmuir adsorption isotherm and chemisorption is the dominant mode of interaction.
5. XPS and FTIR analyses have shown that active functional group of TBA have been adsorbed on the steel surface and led to effective corrosion inhibition of API 5 L X60 in the studied system.
