# Publisher Correction: Eco-friendly 2-Thiobarbituric acid as a corrosion inhibitor for API 5L X60 steel in simulated sweet oilfield environment: Electrochemical and surface analysis studies

**DOI:** 10.1038/s41598-020-59140-x

**Published:** 2020-02-06

**Authors:** Bashir J. Usman, Zuhair M. Gasem, Saviour A. Umoren, Moses M. Solomon

**Affiliations:** 1 Mechanical Engineering Department, King Fahd University of Petroleum and Minerals, Dhahran, Saudi Arabia; 2 Center of Research Excellence in Corrosion, Research Institute, King Fahd University of Petroleum and Minerals, Dhahran, Saudi Arabia

Correction to: *Scientific Reports* 10.1038/s41598-018-37049-w, published online 29 January 2019

This Article contains errors in Reference 23 which was incorrectly given as:

Khodyrev, Y. P. *et al*. Inhibition of Cor-Ten steel corrosion by “green” extracts of Brassica campestris. *Corros. Sci*. **136**, 95 (2018).

The correct reference is listed below:

Casaletto, M. P. *et al*. Inhibition of Cor-Ten steel corrosion by “*green*” extracts of *Brassica campestris*. *Corros. Sci*. **136**, 91 (2018).

